# Comparing trap designs and methods for assessing density of synanthropic flies in Odisha, India

**DOI:** 10.1186/s13071-019-3324-z

**Published:** 2019-02-07

**Authors:** Melissa Bell, Seth Irish, Wolf Peter Schmidt, Soumya Nayak, Thomas Clasen, Mary Cameron

**Affiliations:** 1 Department of Disease Control, London School of Hygiene and Tropical Medicine, Keppel Street, London, WC1E 7HT UK; 2 Xavier Institute of Management, Xavier Square, Jayadev Vihar, Bhubaneswar, Odisha 751013 India; 3 Faculty of Environmental Health, Rollins School of Public Health, Emory University, Clifton Road, Atlanta, Georgia USA; 4 Department of Disease Control, London School of Hygiene and Tropical Medicine, Keppel Street, London, WC1E 7HT UK; 5 Present address: President’s Malaria Initiative and Entomology Branch, Division of Parasitic Diseases and Malaria, Center of Global Health, Centers for Disease Control and Prevention, Atlanta, GA USA

**Keywords:** *Musca domestica*, *Musca sorbens*, Synanthropic flies, Trap design, India

## Abstract

**Background:**

There are many different traps available for studying fly populations. The aim of this study was to find the most suitable trap to collect synanthropic fly populations to assess the impact of increased latrine coverage in the state of Odisha, India.

**Methods:**

Different baits were assessed for use in sticky pot traps (60% sucrose solution, 60 g dry sucrose, half a tomato and an non-baited control), followed by different colours of trap (blue *versus* yellow) and finally different types of trap (baited sticky pot trap *versus* sticky card traps). The experiments were undertaken in a semi-urban slum area of Bhubaneswar, the capital of Odisha. The first experiment was conducted in 16 households over 30 nights while experiments 2 and 3 were conducted in 5 households over 30 nights.

**Results:**

The traps predominantly caught adult *Musca domestica* and *M. sorbens* (78.4, 62.6, 83.8% combined total in experiments 1–3 respectively). Non-baited traps did not catch more flies (median 7.0, interquartile range, IQR: 0.0–24.0) compared with baited traps (sucrose solution: 6.5, 1.0–27.0; dry sucrose: 5.0, 0.5–14.5; tomato: 5.0, 1.5–17.5). However, there were significantly more flies collected on blue sticky pot traps, which caught nearly three times as many flies as yellow sticky pot traps (Incidence Rate Ratio, IRR = 2.91; 95% CI: 1.77–4.79); *P* < 0.001). Sticky card traps (27, 8–58) collected significantly more flies than the non-baited sticky pot traps (10, 1.5–30.5).

**Conclusions:**

Blue sticky card traps can be recommended for the capture of synanthropic fly species as they are non-intrusive to residents, easy to use, readily allow for species identification, and collect sufficient quantities of flies over 12 hours for use in monitoring and control programmes.

## Background

Synanthropic (or filth) flies are commonly found in and around human dwellings [1]. These non-biting flies present a public health problem through their habit of flying between faecal matter and households, facilitating the transmission of enteric diseases by regurgitation, defecation or mechanical transference [1, 2]. Outbreaks of diarrhoeal disease and trachoma are often closely associated with increases in fly numbers, usually during the wet season, and at times when sanitary conditions and hygiene are absent or reduced [3]. Fly control has been found to be protective against the transmission of enteric infections [4], but there is insufficient evidence that the reduction in fly numbers limits disease transmission.

Monitoring synanthropic fly populations can help determine whether programmes that increase latrine coverage are effective. They also give an indication of the specific times when human populations might be at most risk from an increase in diarrhoeal diseases. Fly population monitoring programmes commonly use either sticky cards or baited traps, depending on the purpose and location (external or internal) for sampling populations. Most commonly, sticky cards have been used for indoor populations [5, 6] and baited traps have been utilised for outdoor populations [7, 8]. Monitoring house flies, *Musca domestica*, in household kitchens of rural villages and urban slums is a particular challenge due to the variety of places where the kitchen can be located; indoors or outdoors.

Baited traps allow capture of large numbers of flies and can be classed as a control measure due to the high quantity captured. From the purposes of population density monitoring, these traps allow the identification of species, and monitoring of numbers to measure the effect of control methods on a targeted population. The disadvantage lies in the inability to catch flies individually and prevent flies from contaminating one another for the purpose of testing for bacteria transmission. Baited traps can be too expensive for daily use if needed for continual surveillance performed in multiple villages. Furthermore, their use may be objectionable to residents; baits shown to be attractive for flies, for example, human faeces, rotting vegetation and fish for the capture of *M. domestica* [9, 10], are foul smelling to some people.

The Scudder grill is ideal for providing a measure of the relative abundance of flies in a given area [11, 12]. They can be moved to assess the concentration of flies in different locations. However, grill counts only provide a brief window to assess density of fly populations and are dependent on a variety of factors, such as time of day, weather conditions, user ability, and position of the grill [11, 13, 14].

Sticky cards are relatively cheap, easy to acquire, easy to transport and minimally intrusive to residents [10, 15]. They can be left for weeks at a time if monitoring fly numbers only, for instance on cattle farms in the United States of America [10, 16]. For studies monitoring bacteria in households, they can successfully collect large numbers of flies within 12–24 hours although, the more flies are caught, the ability to trap more flies is reduced as the surface area diminishes [6]. However, it is unknown to what extent the sticky glue could interfere with identification of caught species, or trap dust and other substrates that could hinder the effectiveness of trapping insects and contribute to fly bacterial contamination.

There are many methods that have been used for the capture and assessment of synanthropic flies but there is little uniformity in techniques used [5, 6, 10, 15, 16]. It is known that flies are sensitive to differing wavelengths of light [17] and that varying colours from the spectrum may be used to improve trap catches. Although one study undertaken in the field suggested that colour did not have an effect on trap catches [18], there are several studies that suggest the opposite [16, 19–21]. Hall et al. [22] showed that there can be significant variation in numbers of flies caught on different colours of traps between species as well as within species.

Few recent studies have explored the use of traps to assess species and bacterial carriage as well as population densities but these studies were not conducted on a large scale within houses [23, 24]. Most experiments have either focused on the species and bacterial load or the population density and species.

The objective of the present study was to assess a variety of trapping methods to determine the best design and method for quantifying *M. domestica* densities in household kitchens in order to evaluate the impact of a sanitation randomised control trial on populations of synanthropic flies [25, 26]. This was achieved by: (i) comparing different baits using a sticky pot trap design; (ii) finding the best colour for a sticky card trap; blue and yellow colour sticky card traps; and (iii) comparing the baited pot trap collections with the non-baited sticky card trap method.

## Methods

### Study site

Sampling was undertaken in households in a semi urban slum in Bhubaneswar, Odisha, eastern India (20.27°N, 85.84°E). Latrine coverage in the slum was low and there were many open defecation sites throughout the area: a report in 2008 stated that 77% of households in urban slums throughout Bhubaneswar did not have access to latrine facilities so open defecation is common [27]. Open defecation sites were located in areas of the slum easily accessible by residents and were surrounded by houses. In addition, other sources of faeces, breeding sites for flies, are derived from large numbers of cattle, pigs and chickens that are freely wandering through the slum and surrounding area during the day but were tethered or penned close to the owner’s house at night. The houses within the slums of Bhubaneswar are of mixed construction, either concrete or mud. Trapping was undertaken between July 2011 and April 2012, covering a monsoon, winter and summer season.

### Sampling methods

There were no data relevant to the area on fly abundance and density prior to this study. The sample size required to detect a significant difference between treatment groups was, therefore, calculated based on fly counts obtained from Scudder grills placed in 10 houses over the course of three days, useful for ascertaining numbers although not for detecting differences in species. From the Scudder grill work, assuming an arithmetic mean of 63 and standard deviation (SD) of 47, the sample size was calculated using the formula by Smith, Morrow & Ross to compare the difference between two means [28]. This resulted in a minimum sample size of 57 traps nights per treatment to detect a 66% difference in effect with 80% power and a significance level of 0.05. As the sample size was based on random sampling methodology with a Scudder grill to collect the data, it was decided to increase the trap nights for each treatment group to 150 allowing for a large error margin in the Scudder grill counts when compared to the sticky card or baited pot traps.

### Experimental designs

Each experiment consisted of two different treatments, with the exception of the first experiment, which contained four different treatment groups. Using STATA 11 (Statacorp, USA), a random mixture of 10 households were selected to participate in the second and third experiment, with 16 being chosen for the first experiment. Different households were used for each of the three experiments. The position of traps was randomised around the houses based using a Latin square design; resulting in 4 houses per treatment per night for the first experiment and 5 houses per treatment per night for the second and third experiments. Experiments were conducted over a 30-day period resulting in 120 trap-nights for the first experiment with four different treatment groups and 150 trap-nights for the two subsequent experiments looking at two different treatment groups.

Households were initially mapped and then assigned a number for the purposes of identification and randomisation. Fly traps were set in the kitchen area of a house, often a courtyard area shared by several houses, where preparation and consumption of food usually took place. Traps were set between 10:00 and 12:00 h and collected 24 hours later.

### Experiment 1: determine the best bait to be used in a baited pot trap

**Fig. 1:**
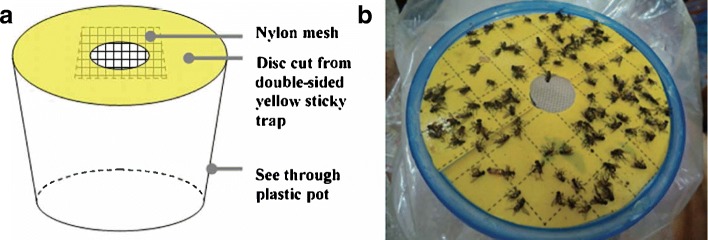
**a** Schematic diagram of the pot trap design. **b** A top-down picture of the pot trap in use. Diagram courtesy of Julie Bristow

Traps were based on a modified version of a pot trap design by Lindsay et al. [29]. A plastic pot (top diameter 150 mm, bottom diameter 100 mm, height 70 mm) with lid (diameter 150 mm) was used to hold the bait and trap the flies. A hole was cut out of the lid (diameter 30 mm) and a circle of nylon mesh (3 mm gauge) attached to the inside of the lid to prevent flies from accessing the bait (Fig. 1a). Yellow sticky card (Product code 10271, Suterra Ltd, Valencia, Spain) was cut to the size and shape of the lid. The card was sticky on both sides; one side was used to attach the card to the lid and one side was used to trap flies (Fig. 1b).

**Fig. 2:**
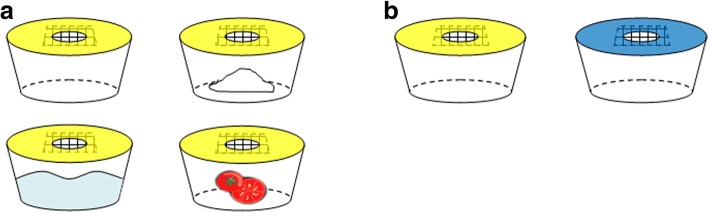
**a** Schematic diagram of the four baited sticky pot traps, showing baits used in the first experiment, clockwise from the top left: control without bait, sugar, half a tomato and sugar water. **b** Schematic diagram showing the different colours, yellow and blue used in the second experiment

Baits were selected based on previous research advocating different types of fruit and vegetables, and sugars that are needed by *M. domestica* for survival and are readily available [7, 9, 30, 31]. The three baits used in the experiment were: (i) no bait (control); (ii) sugar water (60% solution: 60 g of locally available sucrose dissolved in 100 ml tap water); (iii) sugar (60 g of dry sucrose); and (iv) half a tomato (Fig. 2a). The baits were prepared before use and changed daily. The experiment was conducted in the monsoon season, July to August 2011.

### Experiment 2: determine the best colour to use in a sticky card trap

Two colours of sticky card, yellow and blue (Product codes 10271 and 10303, Suterra Ltd, UK) were used as a non-baited fly trap. According to the manufacturers, in all aspects e.g. material type/thickness, dimensions and catch glue, the traps were the same, the only change between the yellow and blue sticky traps was the colour of the base material (Fig. 2b) [32, 33]. The sticky card was placed on pot traps as in the first experiment. The sticky traps were changed daily and flies counted. The experiment was conducted in the dry winter season, November to December 2011.

### Experiment 3: determine the best trap to use, either sticky card traps or baited pot traps

**Fig. 3:**
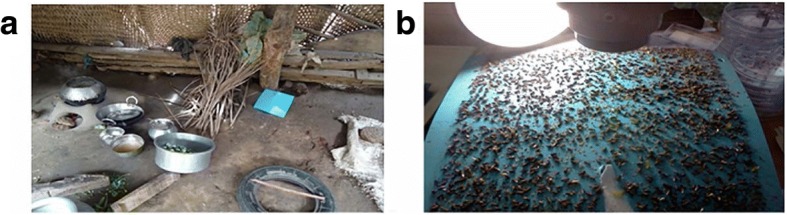
**a** A picture of the sticky card trap in location in a sheltered kitchen area. **b** A picture after trap collection showing the synanthropic flies caught

Baited sticky pot traps, using sucrose solution and blue sticky card, were compared with non-baited blue sticky card traps, each measuring 200 × 245 mm. The baited sticky pot traps were placed on the floor so the horizontal sticky surface was 7 cm from the floor, and the sticky card traps were supported at a 45° angle, using a stick, on the floor with both sticky surfaces exposed to enable capture of flies flying at heights of up to 23 cm from the floor (Fig. 3a, b). The experiment was conducted over the dry summer season, March to April 2012.

### Analysis

Flies were carefully removed from the traps using sterilised forceps, then counted and identified to species using the Fauna of British India series keys: Diptera volume 6: Muscidae; 7: Calliphoridae; and 10: Sarcophagidae [34–36]. Fly densities were analysed using STATA 11 (Statacorp, USA). Data were tested for normality and, if necessary, log-transformed. Data that were skewed, despite log-transformation, were analysed using a negative binomial regression model. The data were over-dispersed and so the untransformed data was analysed using a negative binomial regression model. Total synanthropic fly densities and differences between the main synanthropic species caught, *M. domestica* and *M. sorbens*, also were analysed.

## Results

### Experiment 1: baited pot traps

In total, 1882 flies were captured including 884 *M. domestica* (46.9% of total catch) and 594 *M. sorbens* (31.5% of total catch). No other synanthropic fly species of public health importance was captured during the course of the experiment. However, 34 mosquitoes were collected of which 10 were identified as *Culex quinquefasciatus* and 7 were *Mansonia annulifera.* The remaining 17 could only be identified to the family Culicidae due to glue covering distinguishing marks making further identification impossible. The traps also captured 5 Phlebotominae (Psychodidae). Remains of flies (178 in total) that lacked an abdomen and/or thorax but obviously comprised of at least a pair of wings, head and/or legs were counted towards the final total. It was not possible to identify 27 flies to species, although sufficient characteristics were available to identify the flies as belonging to the family Muscidae. These also were included in the final analysis.

**Fig. 4:**
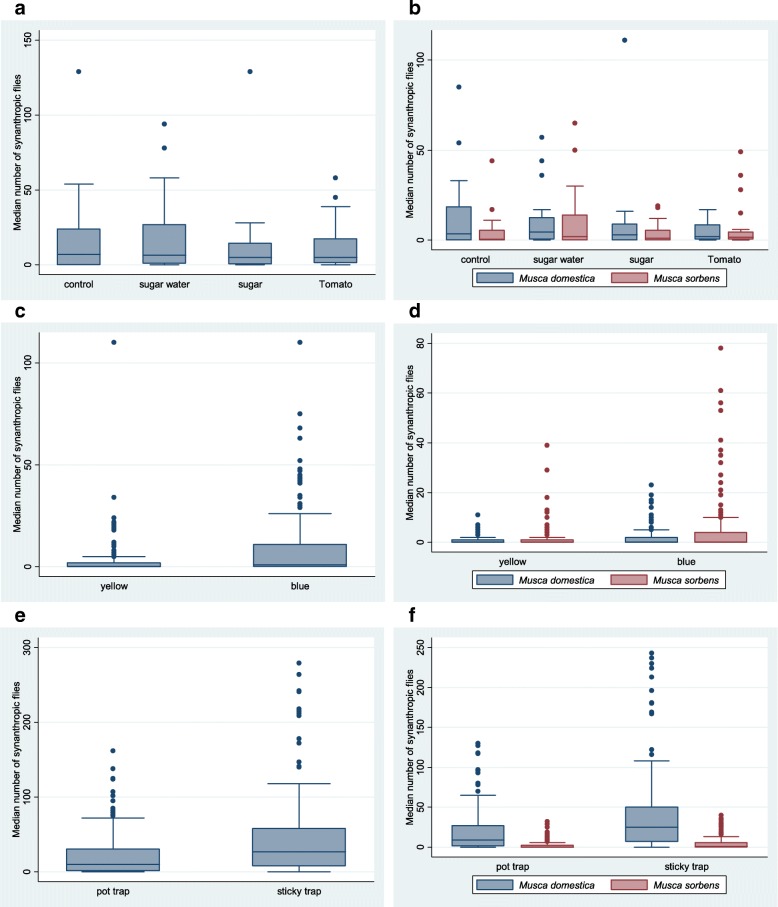
Comparison of traps in experiments designed to find the highest capture rates for synanthropic flies. Median number and interquartile ranges, with outliers, of synanthropic flies, *M. domestica* and *M. sorbens*. **a** Synanthropic flies captured from baited traps compared with control traps (480 trap nights). **b** *M. domestica* and *M. sorbens* captured from the baited experiment (480 trap nights). **c** Synanthropic flies captured from yellow and blue sticky traps (150 trap nights). **d** *M. domestica* and *M. sorbens* captured from yellow and blue sticky traps (150 trap nights). **e** Synanthropic flies captured from pot and sticky traps (150 trap nights). **f** *M. domestica* and *M. sorbens* captured from pot and sticky traps (150 trap nights)

In total, the final analysis included 1478 synanthropic flies and fly remains. The median number of flies collected per trap/night for each treatment group is shown in Table 1. None of the baits used in the experiment caught significantly more flies than the control trap without any bait. Neither were there significant differences between the various baits: sucrose solution *vs* dry sucrose (IRR = 0.65, 95% CI: 0.26–1.59, *P* = 0.341); sucrose solution *vs* tomato (IRR = 0.59, 95% CI: 0.24–1.44, *P* = 0.245); dry sucrose *vs* tomato (IRR = 0.91, 95% CI: 0.37–2.23, *P* = 0.833) (Fig. 4a).

**Table 1:** Comparison of synanthropic flies, *Musca domestica* and *Musca sorbens*, collected from baited pot traps

	*n*	Synanthropic flies (Median, IQR)	Difference (IRR)	95% CI	*P*-value
Control: non-baited pot trap	428	7.0 (0.0–24.0)	Ref.		
Sucrose solution	470	6.5 (1.0–27.0)	1.10	0.45–2.69	0.838
Dry sucrose	304	5.0 (0.5–14.5)	0.71	0.29–1.74	0.455
Tomato	276	5.0 (1.5–17.5)	0.64	0.26–1.58	0.338
*M. domestica*					
Control: non-baited pot trap	320	3.5 (0.0–18.5)	Ref.		
Sucrose solution	235	4.5 (0.5–12.5)	0.73	0.29–1.88	0.519
Dry sucrose	214	3.0 (0.0–9.0)	0.67	0.26–1.71	0.401
Tomato	115	2.0 (0.5–8.5)	0.36	0.14–0.93	0.034
*M. sorbens*					
Control: non-baited pot trap	108	0.5 (0.0–5.5)	Ref.		
Sucrose solution	235	2.0 (0.0–14.0)	2.18	0.77–6.15	0.143
Dry sucrose	90	1.0 (0.0–5.5)	0.83	0.29–2.39	0.734
Tomato	161	1.5 (0.5–4.5)	1.49	0.53–4.23	0.453

*Abbreviations*: *CI* confidence interval, *IQR* interquartile range, *Ref.* reference

When the primary synanthropic species captured were analysed; *M. domestica* were not caught more frequently on the baited sticky pot traps when compared with the control trap (Table 1). The trap containing 60% sucrose solution caught 27% fewer flies than the control trap and the trap containing dry sucrose caught 33% fewer flies. There were 64% fewer flies captured on the traps containing tomato when compared with the control traps (control *vs* tomato; IRR = 0.36, 95% CI: 0.14–0.93, *P* = 0.034). The results for *M. sorbens* were similar to those of *M. domestica*. Baited traps caught similar numbers of *M. sorbens* as the control traps; all of them caught fewer flies, with the exception of 60% sucrose solution baited trap, although this was not significant (control *vs* sucrose solution; IRR = 2.18, 95% CI: 0.77–6.15, *P* = 0.143) (Fig. 4b). When analysed to see whether there were any sex-specific differences between the numbers of flies caught between different traps, the only significant differences detected were between control and tomato traps, where significantly fewer flies were captured for both sexes when analysed separately (*M. domestica* male; control *vs* tomato; IRR = 0.37, 95% CI: 1.48–0.95, *P* = 0.038; *M. domestica* female; control *vs* tomato; IRR = 0.32, 95% CI: 0.11–0.96, *P* = 0.043).

### Experiment 2: yellow *vs* blue as an attractive colour

A total of 2105 flies were caught of which 356 (16.9%) were *M. domestica* and 963 (45.7%) were *M. sorbens*. Nearly twice as many males (64% of the total collection) as females (36% of the total collection) *M. sorbens* were caught. Similarly, more than twice as many *M. domestica* males (68%) were caught when compared with females (32%). Other synanthropic flies caught included *Musca pattoni* (*n* = 92), *Chrysomya megacephala* (*n* = 1) and Sarcophagidae (*n* = 6). Other fly species captured were 140 mosquitoes, predominantly *Culex quinquefasciatus* (*n* = 128) but also *Armigeres kuchingensis* (*n* = 11) and *Aedes albopictus* (*n* = 1), and Phlebotominae (*n* = 2). The remains (wings, head, and legs) of 278 flies were found and identified to the family level (Muscidae). Due to the lack of other distinguishing characteristics, it was not possible to identify to genus or species. These were included in the final analysis. It was not possible to identify 201 flies to species belonging to the family Muscidae due to glue obscuring distinguishing marks, although it was possible to identify the sex. These also were included in the final analysis.

In total, 1890 synanthropic flies were captured over 150 trap nights. The total number of synanthropic flies caught on the yellow traps was 483 (Median, IQR) (0, 0–2) and on the blue traps 1414 (1, 0–11). Blue traps caught almost three times as many flies as the yellow traps (IRR = 2.91; 95% CI: 1.77–4.79, *P* < 0.001) (Fig. 4c).

*Musca domestica* and *M. sorbens* were the dominant synanthropic species caught, comprising 99% of the collection. When these species were analysed by trap, 3.26 more *M. domestica* were caught on the blue traps compared with the yellow traps (Table 2). A similar difference was seen when comparing *M. sorbens* on blue traps and yellow traps (Table 2). For both species, males and females were caught more frequently on blue traps compared with yellow traps (Table 2 and Fig. 4d).

**Table 2:** Comparison of male and female *Musca domestica* and *Musca sorbens* collected from yellow and blue sticky traps

	Total	Total no. (Median, IQR)	Difference (IRR)	95% CI	*P*-value
*M. domestica*					
Yellow trap	94	0 (0–1)	Ref.		
Blue trap	262	0 (0–2)	2.79	1.62–4.80	<0.001
*M. domestica* male					
Yellow trap	57	0 (0–0)	Ref.		
Blue trap	186	0 (0–1)	3.26	1.78–5.97	<0.001
*M. domestica* female					
Yellow trap	37	0 (0–0)	Ref.		
Blue trap	76	0 (0–1)	2.05	1.11–3.79	0.021
*M. sorbens*					
Yellow trap	233	0 (0–1)	Ref.		
Blue trap	730	0 (0–4)	3.13	1.70–5.78	<0.001
*M. sorbens* male					
Yellow trap	148	0 (0–0)	Ref.		
Blue trap	467	0 (0–3)	3.16	1.69–5.89	<0.001
*M. sorbens* female					
Yellow trap	85	0 (0–0)	Ref.		
Blue trap	263	0 (0–1)	3.09	1.55–6.19	<0.001

*Abbreviations*: *CI* confidence interval, *IQR* interquartile range, *Ref.* reference

### Experiment 3: sticky card traps *vs* sucrose baited pot traps

A total of 12,227 flies were caught of which 9161 were *M. domestica* (74.9%) and 1100 were *M. sorbens* (8.9%). Three times as many males as females were caught of both species: for *M. domestica*, 77% were male and 23% were female, and for *M. sorbens*, 75% were male and 25% were female. Other synanthropic flies caught included *Chrysomya megacephala* (*n* = 1), *Stomoxys calcitrans* (*n* = 7) and Sarcophagidae (*n* = 2). Other species of interest captured were Phlebotominae (*n* = 79) and *Culex quinquefasciatus* (*n* = 44). It was possible to identify all flies to family using fly remains (wings, head, and legs) of 887 flies, and these belonged to the family Muscidae but, without any other identifiable characteristics, it was not possible to determine their species. All flies were included in the final count.

In total, 11,158 synanthropic flies were captured, over 150 trap nights. Baited pot traps caught a median of 10 synanthropic flies (IQR: 1.5–30.5) and sticky traps a median of 27 synanthropic flies (IQR: 8–58). Sticky traps caught more than twice the number of synanthropic flies than baited sticky pot traps (IRR = 2.16, 95% CI: 1.59–2.93, *P* < 0.001) (Fig. 4e).

Twice as many *M. domestica,* were caught on the sticky trap than were caught on the baited pot trap. The results were similar when the traps were analysed for *M. sorbens* only. Baited pot traps caught more *M. sorbens* than sticky traps. For both *M. domestica* and *M. sorbens,* twice the number of males and females were caught on the sticky traps as opposed to the baited pot traps (Table 3 and Fig. 4f).

**Table 3:** Comparison of male and female *Musca domestica* and *Musca sorbens* from pot and sticky traps

	Total	Total no. (Median, IQR)	Difference (IRR)	95% CI	*P*-value
*M. domestica*					
Pot trap	2753	9 (1.5–27)	Ref.		
Sticky trap	6408	25 (7–50)	2.19	1.62–2.96	<0.001
*M. domestica* male					
Pot trap	2101	7 (1–18)	Ref.		
Sticky trap	4936	19 (6–39)	2.21	1.64–2.98	<0.001
*M. domestica* female					
Pot trap	652	1 (0–4)	Ref.		
Sticky trap	1472	5 (1–15)	2.12	1.49–3.02	<0.001
*M. sorbens*					
Pot trap	362	0 (0–2.5)	Ref.		
Sticky trap	738	1 (0–6)	1.92	1.24–2.97	0.003
*M. sorbens* male					
Pot trap	263	0 (0–2)	Ref.		
Sticky trap	555	1 (0–5)	1.99	1.27–3.10	0.003
*M. sorbens* female					
Pot trap	99	0 (0–0)	Ref.		
Sticky trap	183	0 (0–1)	1.74	1.02–2.98	0.043

*Abbreviations*: *CI* confidence interval, *IQR* interquartile range, *Ref.* reference

## Discussion

The study helped identify a suitable trap for collecting Muscid flies in India. The best design for trapping synanthropic flies of interest in the transmission of diarrhoeal diseases was a non-baited, blue coloured sticky trap.

The numbers of flies collected during the present experiments were lower than previous studies conducted in animal farms in the USA [10, 16, 37] but comparable to similar field studies conducted in small rural villages in Africa and Asia [38, 39]. Unlike experiments conducted on farms in the USA, where the only breeding, resting, mating sites and source of food is the farm; in Asia and Africa there are many alternative sites that can compete with the baited and sticky traps, such as open defecation sites and rubbish deposits, which may reduce the numbers of flies caught. The unexpected low numbers of flies caught as part of the experiment comparing baits could have contributed towards the lack of difference in fly numbers caught on the baited traps when compared with the non-baited control trap.

There were restrictions regarding the types of baits that could be used for experimentation. For example, although flies are known to be attracted to human faeces [24], it could not be used as an attractant in the baits for cultural sensitivity. Alternative baits, including different meats, fish and chemical attractants [8, 40] were unsuitable for the following reasons: (i) meat, while suitable for smaller studies, would have been difficult to access in high quantities for a larger trial; (ii) fish, while usually available in large quantities, was variable in supply throughout different times of the year; (iii) although commercially produced chemical baits containing imidacloprid or spinosad have been shown to be effective [41–43], the cost associated with buying these and shipping them to India was prohibitive. The lack of any overwhelming stimulus that would attract flies to the trap, distinguishing the traps from alternative local sources and could further explain why there were low numbers of flies caught. In a study by Geden [16], it was recorded that strong olfactory cues often overwhelmed any visual stimuli. In the case of the slum where there were many attractive odours to filth flies present, it is possible that the individual baits were not competitive enough and the sticky trap provided a convenient resting place, despite not having olfactory cues.

During the second experiment comparing different colours of sticky card on a non-baited pot trap, a much higher proportion of *M. sorbens* were captured (46%) when compared with the first (32%) and third experiment (9%). Despite the lack of bait being the key difference between the first and second experiment, it is unlikely that the increase in *M. sorbens* captured was due to the lack of bait which would suggest a possible repellent effect of the baits used. The reason for the increased numbers caught is unknown but probably due to the differences between the houses used to trap the flies, rather than the trap design. When the numbers of flies caught were disaggregated by house, some houses caught substantially more flies than others. This suggests that factors external to the trap in some houses contribute to the increase in fly numbers caught; presence of rubbish or open defecation sites close to the house for example.

Studies that have used some form of coloured trap for capturing synanthropic flies have used either blue [16, 18, 44] or yellow [7, 37, 45] as the attractive colour. It is known that muscid flies are visually sensitive to wavelengths: (i) 490 nm (blue); (ii) 570 nm (yellow); and (iii) 330–350 nm (ultraviolet) [17]. Other species of flies including Calliphoridae, have shown an attraction for wavelengths of darker colours (blue, black, etc.), such as tsetse flies to black and blue targets [46] and stable flies to blue traps [16]. It is possible that the darker blue provides a stronger contrast to the surrounding environment and vegetation than the yellow traps [19, 47]. There was a significant difference in the numbers of muscid flies caught on the blue trap as opposed to the yellow traps, mirroring results seen in an experiment by Diclaro et al. [21]. They found that blue traps were attractive to *M. domestica*, yellow traps were repellent, and blue traps with black lines increased attractiveness.

A previous study had shown that sticky cards are able to capture larger flies than muscids such as Calliphorids [48], and that no flies managed to escape the trap once they had landed on the glued surface. However, personal observation and comments by other researchers, have suggested that the glue on sticky traps is not sufficient and that larger flies can escape even if they land directly on the glue. Despite this, few Calliphorids were observed around houses and the absence of any caught on the traps in the present study is probably due to the lack of those flies around kitchens and households in this area of India in contrast to studies conducted elsewhere in Asia and Africa [24, 39, 49].

Other factors that affected the trap catches included heavy rain. During the second experiment comparing colours of sticky trap, traps placed in outdoor kitchens on days with heavy rain, became soaked and, while the glue was still sticky underneath the water, droplets would form, obscuring the surface. Therefore, although rainfall has been correlated with an increase in fly populations [12, 50, 51], it is possible that trapping success may be reduced during heavy periods of rainfall using sticky traps, if traps are exposed. During the dry season, both summer and winter, dust was present in the kitchens of households that were swept daily. This could result in the partial obscuring of the trap surface reduction in stickiness, and therefore limit the potential number of flies caught.

During the final experiment, comparing a non-baited sticky pot trap with a sticky trap a much larger number of flies were caught. Almost five times as many flies were caught during this experiment than the previous two experiments. One main difference is in the season collected; the final experiment was conducted in the dry summer season just before the advent of the monsoon season. During the monsoon season, collections were possibly not as high as could be expected due to heavy rainfall obscuring the trap. The cooler season could be less conducive to the development of young larvae. In comparison, during the hot season, there is nothing to inhibit or slow population growth, resulting in higher numbers of flies captured.

Another possible reason is the surface area available to catch flies. The surface area of the pot trap was approximately 177 cm^2^; of the sticky trap, 490 cm^2^, almost three times the size of the pot trap. Almost three times the number of flies were caught on the sticky trap when compared with the pot trap. Despite the sticky trap catching significantly more flies, the surface area available to catch flies on the trap could have been responsible for the higher number of flies collected. A larger surface area also provides a larger visual cue.

Throughout each experiment, it was observed that at least double the number of males than females were caught. It is generally assumed that the male: female ratio at emergence is 1:1 [52]. This ratio can be altered by chemicals, factors affecting female survival rates, the position of the trap or the baits used in the trap. This is not the first study to record a bias toward males over females [10, 37] but it is uncommon, as the majority of studies see the reverse [45, 51, 53]. The most likely explanation is the location of the trap was responsible for catching more males than females. The traps were placed inside or adjacent to houses and away from primary breeding sites, such as open defecation areas, animals, abundant oviposition sites. Despite this, the ability to transmit diarrhoeal diseases is not limited to the female alone, unlike other medically important arthropods. Both males and females visit areas where diarrhoeal disease causing bacteria like *Escherichia coli* and *Salmonella* could adhere to the external surface of the fly, to be dislodged when next visiting a human food source [54].

## Conclusions

The non-baited blue sticky card trap collected more flies than the yellow baited sticky pot traps or yellow non-baited sticky card traps. The primary synanthropic fly collected during the third experiment was *M. domestica* and it was captured in greater numbers on the blue sticky card trap compared with the yellow sticky card trap. *Musca sorbens* were collected in high numbers throughout the experiment. While the sticky trap collected larger fly numbers than any other trapping method, there were limitations. The placement of the traps could potentially select for some species over others in comparison with a sweep net method of capture, which can indiscriminately collect species at a variety of locations at differing time points, with the drawback being labour intensive. In field sites such as Odisha, where baited traps might be competing with equally or more attractive odours, non-baited blue sticky card traps provide a strong visual stimulus to induce landing and are a simple way to collect large numbers of synanthropic flies of interest as they are easy to place and less intrusive to the residents than baited traps. Despite the potential limitation of positional bias, these traps could be useful in the context of large monitoring programs to assess fly densities. If combined with an odour attractant that could compete with local odours, this trap could be used as a method of direct fly control using an attract and trap/kill technique, due to the large numbers of flies captured.
